# Spontaneous Intracerebral Hemorrhage Secondary to a Parasagittal Meningioma: A Case Report and Review of the Literature

**DOI:** 10.7759/cureus.46863

**Published:** 2023-10-11

**Authors:** Louis Reier, Christina Mao, Jordan Hough, Fiona Pudewa, Imran Siddiqi, Maxwell A Marino, Anthony Alastra

**Affiliations:** 1 Neurosurgery, Arrowhead Regional Medical Center, Colton, USA; 2 General Surgery, Valley Health System, Las Vegas, USA; 3 Neurology, St. George's University School of Medicine, Whittier, USA; 4 Neurosurgery, California University of Science and Medicine, Colton, USA; 5 Neurosurgery, Riverside University Health System Medical Center, Moreno Valley, USA; 6 Neurosurgery, Desert Regional Medical Center, Palm Springs, USA

**Keywords:** meningioma hemorrhage, bleeding inside meningioma, spontaneous intracerebral hemorrhage, intratumoral hemorrhage, parasagittal meningioma

## Abstract

Meningiomas are the most prevalent tumors within the central nervous system, with most exhibiting benign characteristics. While they are often discovered incidentally, their growth can lead to symptoms such as headaches, visual changes, dizziness, and seizures. Intratumoral hemorrhage (ITH) within meningiomas is a rare occurrence. This phenomenon carries a poor prognosis, as evidenced by significant rates of morbidity and mortality. This case report describes a unique case of a 52-year-old male who experienced a spontaneous right parietal lobe intracerebral hemorrhage adjacent to the superior sagittal sinus. Subsequent investigations revealed this to be an ITH due to an underlying WHO-grade I meningioma. This case emphasizes that while ITH in meningiomas is rare, prompt recognition and surgical intervention ensure optimal patient outcomes.

## Introduction

Meningiomas are extra-axial tumors that originate from arachnoid cap cells. They are the most common type of tumor found within the central nervous system, accounting for roughly 37% of primary central nervous system tumors [1]. Most harbor benign histopathological features; however, roughly 10% have atypical or malignant features. The incidence is twice as likely for females and has been linked to ionizing radiation and genetic mutations in the neurofibromatosis 2 gene [2]. The majority of meningiomas found on radiographic images are incidental; however, symptoms may begin to present as the tumor grows and causes a local mass effect. While presenting symptoms largely depend on location and size, the most common presenting symptoms reported in the literature are headaches, visual changes, dizziness, and seizures [1].

Spontaneous hemorrhage occurring within a brain mass, also known as intratumoral hemorrhage (ITH), is a rare entity, occurring in an estimated 1.4% to 10% of all intracranial tumors [3]. This phenomenon is most commonly seen in pituitary tumors, high-grade gliomas, and metastatic brain tumors (highest incidence when the primary source originates from renal cell carcinoma, melanoma, thyroid carcinoma, or choriocarcinoma) [4]. Although meningiomas are highly vascularized tumors, associated ITH is extremely rare, occurring in only 0.5% to 2.4% of cases [3]. The presence of ITH within a meningioma portrays a poor prognosis, with a morbidity of 36% and mortality of 21.1% [5].

The most common locations of meningiomas associated with spontaneous ITH are thought to be intraventricular and convexity [6]. The most common histological subtypes are thought to be fibrous, angioblastic, and malignant [6]. However, this is based on data obtained from several case series, and high-quality research on specific characteristics portraying high-risk features of ITH is lacking given the limited number of cases reported. Here, we present the case of a 52-year-old male who presented with a spontaneous right parietal lobe intracerebral hemorrhage (ICH) adjacent to the superior sagittal sinus, which was the result of an ITH within an underlying meningioma (WHO grade I).

## Case presentation

A 52-year-old right-handed Caucasian male presented to the hospital with a chief complaint of occipital headaches starting three days prior to evaluation. His headaches were intermittent, fluctuating between 5-8/10 in severity, and dependent upon activity. For example, two days prior to the presentation, he noticed the severity of his headaches dramatically worsened while exercising but resolved 15 minutes after he stopped exercising. He described the pain quality as "sharp" and "stabbing." He stated that the location of his pain is constant, pointing to the exact location overlying the tumor. He denied nausea, vomiting, changes in vision, seizure-like activity, focal weakness, numbness, tingling, or any type of abnormal sensation.

He denied having any medical conditions or prior surgeries. He took no home medications (including over-the-counter), had no allergies, and smoked marijuana, but denied tobacco or alcohol use. He denied having any family members with a history of bleeding disorders or cancer. A complete and thorough neurological exam was conducted, and the only deficit appreciated was a mild upgoing drift in his left upper extremity. Otherwise, his Glasgow coma score (GCS) was 15, he was alert and oriented x4, cranial nerves II-XII were intact (which included a thorough visual field examination), and on formal motor evaluation, he had 5/5 strength throughout all muscle groups of the upper and lower extremities.

Imaging on presentation

**Figure 1:**
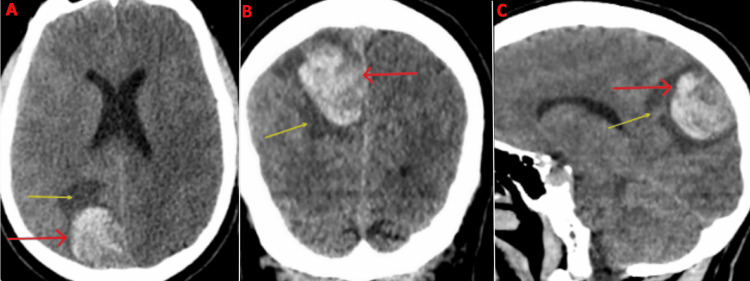
CT head on arrival to the emergency department with views of the axial (A), coronal (B), and sagittal (C) planes Red arrows: Hyperdense mass, Yellow arrows: Vasogenic edema

**Figure 2:**
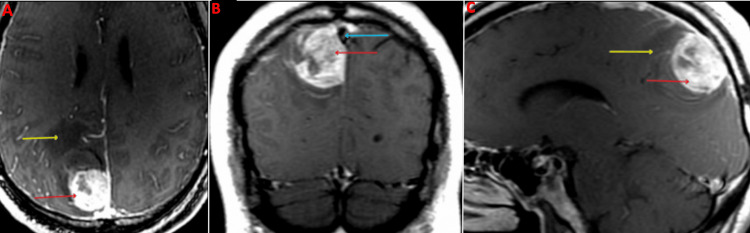
Preoperative MRI brain post contrast with views of the axial (A), coronal (B), and sagittal (C) planes Red arrows: Mass, Blue arrow: Superior sagittal sinus, Yellow arrows: Vasogenic edema

**Figure 3:**
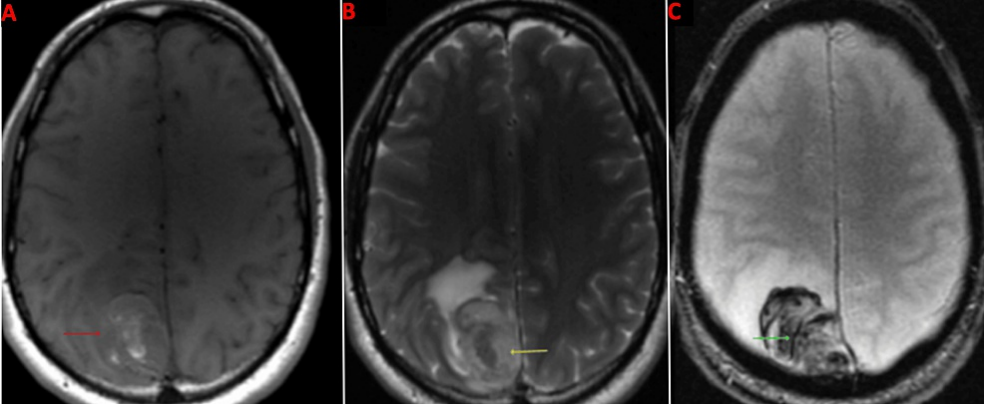
Preoperative MRI brain in the axial plane A: Mass is isointense to the brain on T1 weighted image (red arrow), B: Mass is hyperintense to the brain on T2 weighted image (yellow arrow), C: Mass is hypointense to the brain on GRE (green arrow) sequence GRE: Gradient echo

**Figure 4:**
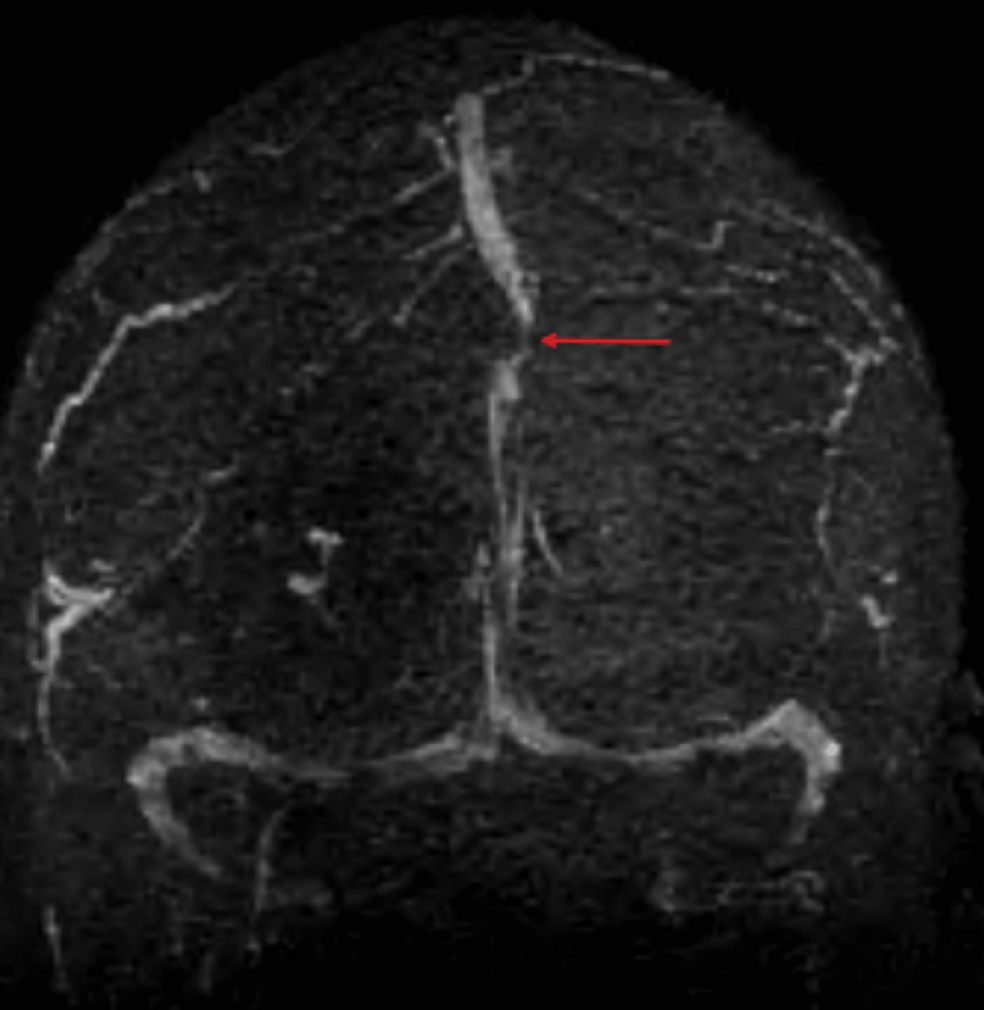
Preoperative MRV The superior sagittal sinus is severely stenotic, although patent (red arrow), and displaced to the left of the midline. MRV: Magnetic resonance venography

Upon arrival at the emergency department, a CT head without contrast demonstrated a hyperdense, well-circumscribed lesion in the right parietal lobe measuring 3.2 cm in length, 1.6 cm in width, and 3.2 cm in height with surrounding vasogenic edema (Figure 1). An MRI brain with and without contrast was subsequently ordered and demonstrated a right parietal parasagittal homogeneously enhancing mass abutting the meninges in the interhemispheric fissure measuring 3.7 cm in length, 3 cm in width, and 3.8 cm in height, with surrounding vasogenic edema causing a mild mass effect on the posterior aspect of the superior sagittal sinus (Figure 2). The mass was isointense to the brain on T1 weighted imaging, hyperintense to the brain on T2 weighted imaging, and hypointense to the brain on the gradient echo (GRE) sequence, all consistent with hyperacute blood (Figure 3). For preoperative planning, a magnetic resonance venography (MRV) was ordered to assess the patency of the superior sagittal sinus (Figure 4). A CT chest, abdomen, and pelvis with and without contrast was also completed as part of the patient’s metastatic workup and no lesions were identified. 

Treatment and surgical resection

We recommended a semi-urgent surgical resection. Our rationale for doing surgery on a semi-urgent basis was based on several key factors. First, the posterior aspect of the patient’s superior sagittal sinus was severely stenotic secondary to the mass effect of the tumor (as seen above in Figure 4). If the mass continued to grow, it could potentially completely occlude the SSS and ultimately put the patient at high risk of a catastrophic stroke. Second, the tumor was neighboring the sensory and motor cortices and producing vasogenic edema that already involved both of these eloquent cortices (which explains why the patient had an upgoing left upper extremity drift on the exam). Further tumor growth could potentially lead to left-sided paresis and sensory abnormalities. And finally, our last reason for recommending surgery was to establish a diagnosis. Given that most brain tumors that spontaneously hemorrhage are high-grade gliomas or metastases, we felt it was important to establish a diagnosis in a timely manner in case the patient needed further oncology treatments such as chemotherapy or radiation.

**Figure 5:**
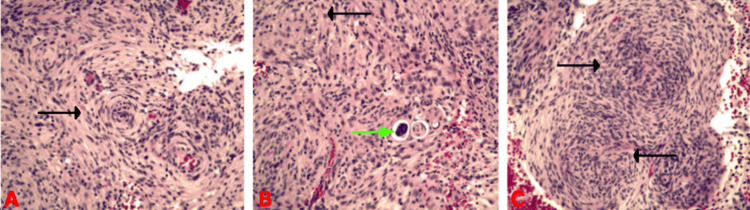
Histology of the patient’s WHO grade 1 meningioma with hematoxylin and eosin stain Spindle cells in a whorled pattern can be seen in sections A, B, and C (black arrows). The psammoma body can be seen in section B (green arrow).

The patient consented to surgery and was taken to the operating room the next day. Given the proximity of the tumor to the motor and sensory cortices, we requested intraoperative neuromonitoring (IONM), specifically the following modalities: somatosensory evoked potentials (SSEPs), motor evoked potentials (MEPs), and electromyography (EMG). The patient was positioned supine, head turned to the left, allowing for the right parietal lobe mass to be at the highest point of the operative field. A straight, linear incision perpendicular to the patient's SSS was made. Stealth navigation (Medtronic Minimally Invasive Therapies, Minneapolis, MN, USA) was used to mark the boundaries of the tumor, and the craniotomy was performed. The dura was opened in a 'U' shape with the base tacked up and reflected over the SSS. We then proceeded with dissection. Immediately after penetrating the tumor capsule, a large, black-colored blood clot was encountered with surrounding yellow-tinged granular tissue. The tumor immediately started bleeding profusely and continued to do so until the last portion of the tumor was resected. Pathology came back as WHO grade-I meningioma, and histology sections are shown below in Figure 5.

Postoperative imaging 

**Figure 6:**
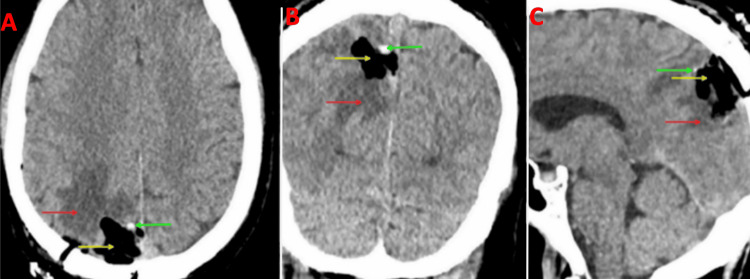
Postoperative CT head with views of the axial (A), coronal (B), and sagittal (C) planes demonstrating interval postoperative changes of right parietal-occipital craniotomy for resection of previously visualized mass Red arrows: Persistent vasogenic edema in the right parietal-occipital lobes, Yellow arrows: Postoperative pneumocephalus, Green arrows: Tiny amount of blood in the operative bed No mass, hemorrhage, or hydrocephalus are seen. No major vessel vascular territory infarct is seen. No intra or extra-axial fluid collection is seen.

**Figure 7:**
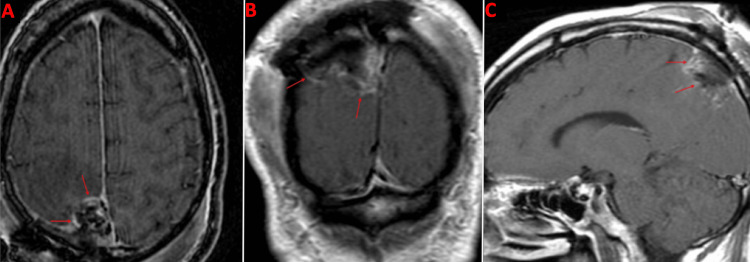
Postoperative MRI brain post contrast in the axial (A), coronal (B), and sagittal (C) planes Red arrows: Peripheral enhancement is seen at the surgical cavity which most likely represents Surgicel

A CT head without contrast was obtained immediately following surgery, demonstrating expected postoperative changes (Figure 6). Twenty-four hours after surgery, the patient obtained his postoperative MRI scan (Figure 7), which again showed typical postoperative changes as well as peripheral enhancement at the surgical cavity, which likely represents Surgicel (Ethicon Inc., Cincinnati, OH, USA) used to line the tumor cavity following resection for hemostasis.

Postoperative clinical course

The patient tolerated surgery well without any changes in neuromonitoring. He was extubated immediately after surgery, and shortly after, he was back to his baseline neurological status. He was admitted to the ICU for neuromonitoring overnight. The next day (postop day one), he was downgraded out of the ICU. He worked with physical therapy (PT) and was ambulatory without assistance. On postop day two, PT recommended he return home to his prior living situation, and he was discharged from the hospital the following day (postop day three). When he left the hospital, he was back to functioning at his neurological baseline. At his third-month follow-up appointment, surveillance MRI showed no signs of tumor recurrence.

## Discussion

Meningiomas are the most common primary brain tumor, accounting for approximately one-third of all primary brain tumors. Most are found incidentally on radiographic images obtained for other reasons. Patients are usually asymptomatic and can be managed conservatively with observation. However, if patients present with symptoms attributable to the meningioma or tumor growth seen on serial imaging, then surgical intervention is typically indicated [2].

Spontaneous ITH primarily occurs in the setting of high-grade gliomas and metastatic brain tumors, but rarely with meningiomas. Intratumoral hemorrhage within meningiomas constitutes a mere 1.3% to 2.4% of all cases [7-9]. When this does occur, it is most common in convexity and paraventricular locations [5]. We presented a unique case of ITH within a benign WHO grade I parasagittal meningioma, offering an opportunity to explore the presentation, risk factors, pathophysiology, management strategies, prognosis, as well as other unique features of this rare clinical entity. We performed an extensive literature search and identified 10 cases of parasagittal ITH meningiomas reported in the past decade, and details about each of these cases are listed in Table 1. 

**Table 1:** Reported cases of parasagittal meningioma intratumoral hemorrhage Listed above is a summary of cases reported in the past decade of parasagittal meningiomas presenting as spontaneous ITH. The average tumor size was 4.61 cm, with a male predominance. Around 44.4% of cases involved WHO grade II or III meningiomas, and symptom onset was sudden in 90% of patients. While 40% of patients made a full recovery, 30% perished. ITH: Intratumoral hemorrhage

Article		Study type (case report, case series, etc.)	Patient age	Patient sex	Tumor size and location	Tumor histology	WHO grade	Symptom onset	Surgery or no surgery?	Outcome	
Byard et al. [10]		Case report	46	M	6 x 7.5 cm right frontal parasagittal	Fibrous	WHO I	Sudden	None (expired)	Expired	
Xie et al. [11]		Case series	45	M	3.9 cm, parasagittal	Atypical meningioma	WHO II	Sudden	Surgery	Tumor recurrence	
		Case series	46	F	3.1 cm, parasagittal	Microcystic meningioma	WHO I	Sudden	Surgery	Full recovery	
		Case series	46	F	2.1 cm, parasagittal	Angiomatous meningioma	WHO I	Sudden	Surgery	Full recovery	
		Case series	79	M	6.8 cm, parasagittal	Malignant meningioma	WHO III	Sudden	Surgery	Expired	
											Case series	64	M	4.1 cm, parasagittal	Fibrous meningioma	WHO I	Sudden	Surgery	Full recovery	
		Wang et al. [8]		Case series	45	M	3.9 cm, parasagittal	Atypical meningioma	WHO II	Sudden										None (expired)	Expired	
				Case series	64	M	4.1 cm, parasagittal	Fibrous meningioma	WHO I	Sudden	Surgery	Neurological deficit	
Hu et al. [12]													Case report	58	M	6.0 cm, parasagittal	N/A	N/A	Sudden	Surgery	Full recovery	
Birua et al. [13]		Case series	19	F	Parasagittal (size not stated)	Rhabdoid meningioma	WHO III	Gradual	Surgery	NA	
Study statistics			Mean age: 51.2, Median: 46, Mode: 46	Male to female ratio (M:F) 7:3	Size (only 9 listed): mean: 4.61; median: 4.1; mode: 3.9, 4.1; min: 2.1; max: 7.5	3 fibrous, 2 atypical, 1 microcystic, 1 angiomatous, 1 rhabdoid, 1 malignant, 1 not listed	WHO I: 5, WHO II: 2, WHO III: 2, Not available: 1	Symptom onset sudden: 9, Gradual: 1	Surgery versus no surgery: 8 underwent surgery and 2 didn't because of poor prognosis/death soon after the hemorrhage occurred.	Expired: 3, Full recovery: 4, Neuro deficit: 1, Tumor reoccured: 1, Not reported: 1	

Presentation

Typical presenting signs and symptoms of ITH in meningiomas include sudden onset of stroke-like symptoms, rapid onset of headache, nausea and vomiting, neurological disturbances, loss of consciousness, seizures, and coma [7,11]. However, symptoms can vary based on the anatomical location affected. Our patient experienced significant symptom exacerbations during periods of strain.

Risk factors

Suggested risk factors reported in the literature for meningiomas presenting in the form of spontaneous hemorrhage include age (>70 or <30), history of traumatic brain injury, antiplatelets or anticoagulant use, hypertension, serotonin modulating medications, and estrogen replacement hormone therapy [5,14]. Interestingly, our patient did not have any of these risk factors. 

Pathophysiology

The rarity of ITH in meningiomas could be attributed to the fact that they are typically slow-growing tumors with a rich fibrous matrix that potentially provides structural stability, reducing the chances of hemorrhage. Thus, the pathophysiology leading to hemorrhage in these cases remains unclear, although several theories have been suggested (Table 2). 

**Table 2:** Theories of pathophysiology behind hemorrhage occurrence within meningiomas ITH: Intratumor hemorrhage

Article	Theory
Sinurat et al. [3]	Suggests that rapid tumor growth, stretching of adjacent bridging veins, and rupture of defective intratumoral blood vessels may play a role.
Wang et al. [8]	Used tumor markers to determine the different types of blood vessels in hemorrhagic meningiomas. They reported that meningioma vasculature is heterogenous, consisting of differentiated and undifferentiated blood vessels. Their study discovered an increased number of undifferentiated vessels in hemorrhagic meningioma cases, suggesting the correlation.
Alnaami et al. [15]	Discuss the incidence of aneurysm associated meningiomas with subsequent hemorrhage. They report two significant theories that may increase the likelihood of ITH. First, meningiomas are known to be highly vascularized. The increased pressure from blood flow into the tumor may increase the risk of aneurysm formation and then subsequent rupture. Second, adhesions from tumor inflammation could potentially cause damage to the vessel wall, increasing the risk of aneurysm formation and subsequent rupture.

Management strategies and prognosis

Early diagnosis of ITH meningiomas is crucial, as definitive surgical resection is associated with the best possible outcome, lowering the mortality rate from 55% to 13% [7,16]. Furthermore, in a case series by Leclerc et al., the re-hemorrhage rate among those who did not undergo surgery was 74%, occurring on average just 120 days after their initial presentation [17]. This high re-hemorrhage rate underscores the importance of surgical resection during the current hospitalization, barring any medical comorbidities that preclude surgery.

## Conclusions

This case highlights an unusual presentation of a parasagittal meningioma presenting as spontaneous ITH, a phenomenon infrequently reported in the literature. Key learning points include the following: surgical resection substantially lowers morbidity and mortality; without surgery, the re-hemorrhage rate is exceedingly high; and gross total resection (GTR) is the best method for obtaining hemostasis. We attribute the successful management of our patient to prompt surgical intervention. Although no specialty guidelines exist, we suggest prompt surgical resection be considered the standard of care.

## References

[1] Bhala S, Stewart DR, Kennerley V, Petkov VI, Rosenberg PS, Best AF (2021). Incidence of benign meningiomas in the United States: current and future trends. JNCI Cancer Spectr.

[2] Wiemels J, Wrensch M, Claus EB (2010). Epidemiology and etiology of meningioma. J Neurooncol.

[3] Sinurat R, Banjarnahor JD (2017). Incidental bleeding meningioma. Asian J Neurosurg.

[4] Ostrowski RP, He Z, Pucko EB, Matyja E (2022). Hemorrhage in brain tumor — an unresolved issue. Brain Hemorrhages.

[5] Bosnjak R, Derham C, Popović M, Ravnik J (2005). Spontaneous intracranial meningioma bleeding: clinicopathological features and outcome. J Neurosurg.

[6] Pressman E, Penn D, Patel NJ (2020). Intracranial hemorrhage from meningioma: 2 novel risk factors. World Neurosurg.

[7] Huang RB, Chen LJ, Su SY (2021). Misdiagnosis and delay of diagnosis in hemorrhagic meningioma: a case series and review of the literature. World Neurosurg.

[8] Wang HC, Wang BD, Chen MS, Li SW, Chen H, Xu W, Zhang JM (2016). An underlying pathological mechanism of meningiomas with intratumoral hemorrhage: undifferentiated microvessels. World Neurosurg.

[9] Matsuoka G, Eguchi S, Ryu B, Tominaga T, Ishikawa T, Yamaguchi K, Kawamata T (2019). Treatment strategy for recurrent hemorrhage from meningioma: case report and literature review. World Neurosurg.

[10] Byard RW (2017). Parasagittal meningioma: a not so benign entity. Med Sci Law.

[11] Xie ZR, Wang HC, Tong YL, Li SW, Chen MS, Wang BD (2022). Radiological classification of meningiomas with hemorrhagic onset and its clinical significance. Oncol Lett.

[12] Hu S, Zhang Y, Sun Y (2018). Lung metastases from intracranial bleeding meningioma: a case report. Medicine (Baltimore).

[13] Birua GJ, Sadashiva N, Konar S (2021). Rhabdoid meningiomas: clinicopathological analysis of a rare variant of meningioma. Clin Neurol Neurosurg.

[14] Abolfotoh M, Brzezicki G, Fiester P, Tavanaiepour D (2021). A rare case of life-threatening multicompartmental spontaneous intracranial hemorrhage from a grade 1 convexity meningioma. Cureus.

[15] Alnaami I, Ho P, Lu JQ, Wheatley B (2013). Case report: meningioma with intra-tumoural haemorrhage secondary to ruptured distal anterior cerebral artery aneurysm. Open Neuroimag J.

[16] Cheng MH, Lin JW (1997). Intracranial meningioma with intratumoral hemorrhage. J Formos Med Assoc.

[17] Leclerc A, Gohel H, Malczuk J, Anzalone L, Emery E, Gaberel T (2023). Systematic review of meningiomas revealed by spontaneous intracranial hemorrhage: clinicopathological features, outcomes, and rebleeding rate. World Neurosurg.

